# Ligands for Pheromone-Sensing Neurons Are Not Conformationally Activated Odorant Binding Proteins

**DOI:** 10.1371/journal.pbio.1001546

**Published:** 2013-04-30

**Authors:** Carolina Gomez-Diaz, Jaime H. Reina, Christian Cambillau, Richard Benton

**Affiliations:** 1 Center for Integrative Genomics, Faculty of Biology and Medicine, University of Lausanne, Lausanne, Switzerland; 2 Architecture et Fonction des Macromolécules Biologiques, UMR 7257 CNRS and Aix-Marseille University, Marseille, France; Texas A&M Health Science Center, United States of America

## Abstract

Pheromones form an essential chemical language of intraspecific communication in many animals. How olfactory systems recognize pheromonal signals with both sensitivity and specificity is not well understood. An important in vivo paradigm for this process is the detection mechanism of the sex pheromone (*Z*)-11-octadecenyl acetate (*cis*-vaccenyl acetate [cVA]) in *Drosophila melanogaster*. cVA-evoked neuronal activation requires a secreted odorant binding protein, LUSH, the CD36-related transmembrane protein SNMP, and the odorant receptor OR67d. Crystallographic analysis has revealed that cVA-bound LUSH is conformationally distinct from apo (unliganded) LUSH. Recombinantly expressed mutant versions of LUSH predicted to enhance or diminish these structural changes produce corresponding alterations in spontaneous and/or cVA-evoked activity when infused into olfactory sensilla, leading to a model in which the ligand for pheromone receptors is not free cVA, but LUSH that is “conformationally activated” upon cVA binding. Here we present evidence that contradicts this model. First, we demonstrate that the same LUSH mutants expressed transgenically affect neither basal nor pheromone-evoked activity. Second, we compare the structures of apo LUSH, cVA/LUSH, and complexes of LUSH with non-pheromonal ligands and find no conformational property of cVA/LUSH that can explain its proposed unique activated state. Finally, we show that high concentrations of cVA can induce neuronal activity in the absence of LUSH, but not SNMP or OR67d. Our findings are not consistent with the model that the cVA/LUSH complex acts as the pheromone ligand, and suggest that pheromone molecules alone directly activate neuronal receptors.

## Abstract 2

An *in vivo* functional analysis of an odorant binding protein in *Drosophila* challenges the established model of its role in pheromone signaling.

## Author Summary

Animals produce chemical signals, called pheromones, to communicate with other members of the same species to regulate social behaviors. How pheromones are detected is a fundamental question. One important model system for pheromone recognition is in *Drosophila*, where the sex pheromone *cis*-vaccenyl acetate (cVA) activates olfactory sensory neurons expressing the odorant receptor OR67d. cVA neuronal stimulation requires not only OR67d but also an extracellular odorant binding protein called LUSH. Previous work showed that cVA association with LUSH induces a conformational change in the protein. A mutant version of LUSH that mimics this structural alteration—when expressed in vitro and infused around OR67d neurons—stimulates these neurons in the absence of cVA, suggesting that the ligand that activates OR67d neurons is a cVA/LUSH complex rather than free cVA. By contrast, we show that the same mutant, when expressed in vivo, does not activate OR67d neurons, questioning the significance of the conformational change. We also show that high concentrations of cVA can induce neuronal activity in the absence of LUSH, but not OR67d, indicating that LUSH is important but not essential for pheromone detection. Together, these findings challenge the established model of cVA signaling and suggest that this pheromone directly activates OR67d.

## Introduction

Pheromones—“carriers of excitement” in Greek—are chemical signals produced by an individual and recognized by conspecifics to induce a defined behavioral response [1]. Because pheromones are the basis of communication controlling diverse social interactions in many organisms (e.g., aggregation, kin recognition, sexual and competitive behaviors), understanding how these chemicals are detected has long been a fundamental question [1].

Many animal pheromones are long-chain hydrocarbons [2], whose structural versatility provides a large chemical repertoire for the generation of species- and behavior-specific signals [3],[4]. In mammals, pheromones can also be peptides, such as rodent Exocrine gland-secreting peptide 1, which is released in male tears and enhances female sexual receptivity [5],[6], or small proteins, such as major urinary proteins [7].

**Figure 1:**
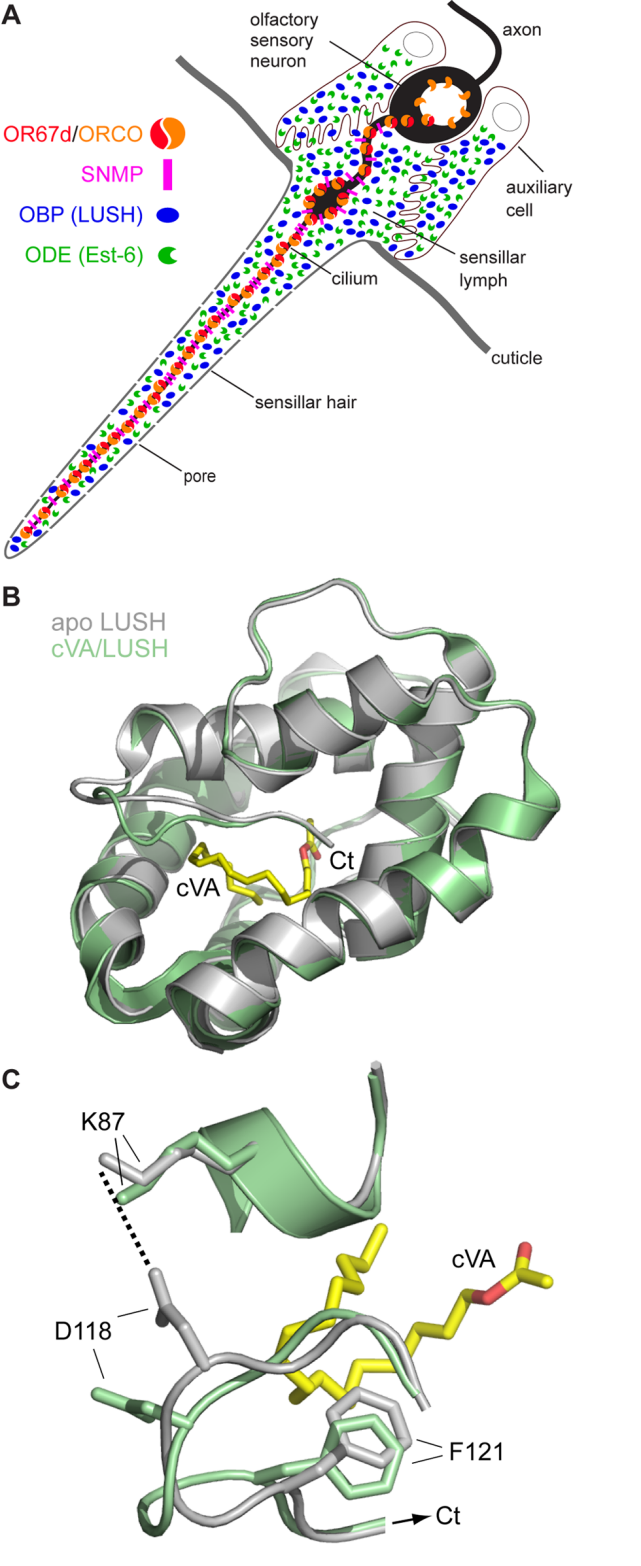
Pheromone-dependent conformational changes in LUSH. (A) Schematic of a pheromone-sensing trichoid sensillum illustrating the major ultrastructural features and proteins involved in detection of cVA. The OSN bears a single sensory cilium where the heteromeric pheromone receptor OR67d/ORCO and the CD36-related SNMP are localized. Auxiliary cells secrete at least three OBPs, including LUSH, and ODEs, including the carboxylesterase Est-6 [49], into the lymph that bathes the OSN cilium within the sensillar hair lumen. (B) cVA-dependent conformational changes in LUSH. Ribbon view of the superimposed backbones of apo LUSH (grey) and cVA/LUSH (green, monomer A, conformation A only is represented; see also Figure S2) (PDB IDs 1T14 [27] and 2GTE [29], respectively). The ligand, cVA, is depicted in stick form (yellow, carbon atoms; red, oxygen atoms). The most prominent conformational differences between the structures are within the C-terminal tail (Ct). (C) Close-up of the regions of LUSH corresponding to residues 83–87 and 115–123 for the structures shown in (B). The side chains of K87, D118, and F121 are represented by sticks. In apo LUSH (grey)—but not in this conformation of cVA/LUSH (green)—K87 and D118 can form a salt bridge (dotted line) (see also Table 1 and Figure S2).

Pheromone detection has been intensively studied in insects [8]–[12]. These volatile chemicals are captured from the air on the surface of insect antennae, head appendages that are covered with porous, cuticular sensory hairs, called sensilla. Pheromone molecules pass, probably by diffusion [13], through the pores to the interior of sensilla, which house the ciliated dendritic endings of olfactory sensory neurons (OSNs) (Figure 1A). These cilia are bathed in lymph fluid rich in odorant binding proteins (OBPs) (also known as pheromone binding proteins [PBPs]) and odorant degrading enzymes (ODEs), which are secreted from auxiliary cells that flank OSN somata. Three transmembrane receptors localize to pheromone-sensing OSN cilia: one member of the odorant receptor (OR) repertoire, which is thought to be the principal determinant of pheromone response specificity [14],[15], the co-receptor ORCO [16],[17], and a CD36-related protein called sensory neuron membrane protein (SNMP) [18]–[21].

One of the best-studied pheromone detection systems is that of the *D. melanogaster* sex pheromone (*Z*)-11-octadecenyl acetate (*cis*-vaccenyl acetate [cVA]), which evokes neural activity in OR67d-expressing OSNs (Figure 1A). Genetic analyses demonstrated that cVA detection requires OR67d [22],[23], ORCO [20],[21], and SNMP [20],[21], as well as the OBP LUSH [24]. Unexpectedly, loss of LUSH also results in decreased spontaneous firing of OR67d neurons—in the absence of pheromone—leading to the proposition that this OBP plays a direct role in cVA-evoked neuronal activity, rather than acting simply as a solubilizer or carrier of this pheromone [24].

In vitro, LUSH binds cVA as well as several short-chain *n-*alcohols [25]–[27], and phthalates [28]. X-ray crystal structures for apo (unliganded) LUSH, various alcohol/LUSH complexes, and the cVA/LUSH complex have been reported [25]–[27],[29]. A notable common feature of complexes of LUSH with either cVA or alcohols is that the ligand is almost completely encapsulated within the six α-helical bundle—similar to many other ligand/OBP complexes [30],[31]—making it unlikely for ORs to be able to recognize directly the ligand within this complex (Figure 1B) [29]. Importantly, the cVA/LUSH complex is structurally distinct from apo LUSH or alcohol/LUSH complexes [25]–[27],[29], in particular within the C-terminal tail (amino acids 113–124 in the processed LUSH protein) (Figure 1B and 1C) [29]. These observations led to the suggestion that this unique conformation of the cVA/LUSH complex is important for the stimulation of OR67d neurons [29].

The functional significance of these cVA-evoked structural changes has been tested by recombinant expression of site-directed mutant versions of LUSH that are predicted to exhibit different degrees of this conformational change [29]. These purified proteins were infused into individual sensilla housing OR67d OSNs in *lush* mutant animals, which lack the endogenous OBP, via a glass recording electrode. Basal and cVA-evoked activity of these neurons was then measured by extracellular electrophysiological recordings. A complementary pair of mutants, LUSH^F121A^ and LUSH^F121W^, predicted to diminish or enhance the conformational change, respectively, led to decreased or increased cVA sensitivity. Notably, one mutant, LUSH^D118A^, which disrupts a predicted salt bridge suggested to be present in apo but not cVA-bound LUSH [29], induced increased firing of OR67d neurons in the absence of cVA. Presentation of cVA did not further increase this neuronal activation. These observations led to a model in which cVA induces “conformational activation” of LUSH, and that it is this cVA/LUSH complex—and not free cVA—which is recognized (through an undefined mechanism) by the neuronal pheromone receptors [29].

This model contrasts with the widely held idea that pheromone molecules must ultimately directly bind and stimulate pheromone-sensing ORs [10],[11],[14],[15], although pheromone/OR interactions have never been shown biochemically. However, the role of an extracellular protein, LUSH, in pheromone neuron activation in insects provided an interesting parallel with the discovery that small protein pheromones can stimulate olfactory neurons in mammals [7].

We have tested this model [29] by transgenic expression of the same LUSH mutants predicted to affect the cVA-induced conformational changes. We find that in vivo-expressed LUSH mutants do not recapitulate the effects observed with recombinant LUSH. We also show that LUSH, but not SNMP or OR67d, is dispensable for pheromone-evoked activity at high cVA concentrations. These results do not support the proposition of the cVA/LUSH complex as the pheromone-sensing neuron ligand.

## Results

### A Transgenic System for Structure-Function Analysis of LUSH

**Figure 2:**
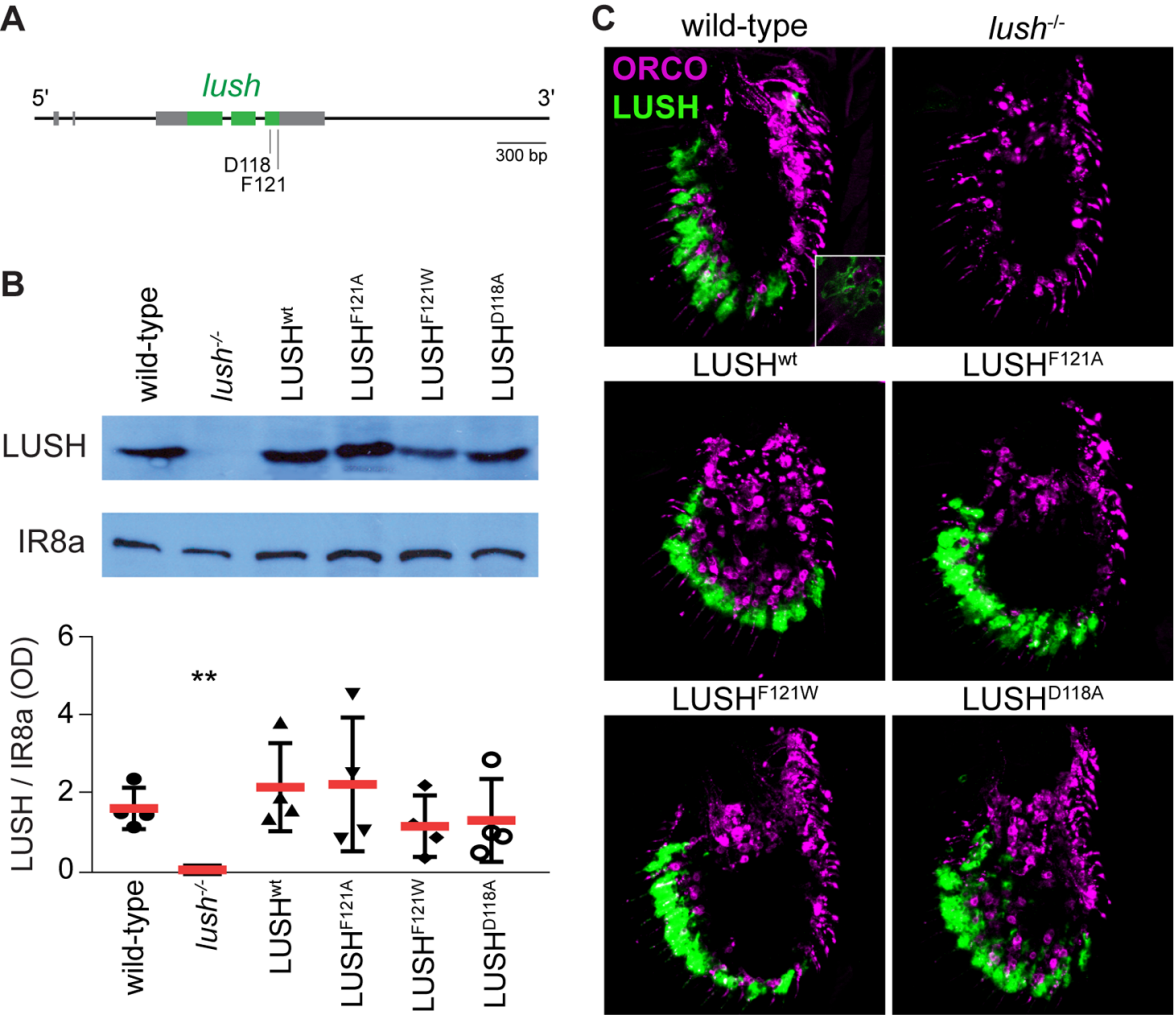
A transgenic system for structure-function analysis of LUSH. (A) Schematic representation of the *lush* genomic rescue construct. Coding exons are colored green and 5′- and 3′-UTRs in grey; introns and flanking genomic DNA are represented by a black line. Each transgene was inserted at the same genomic location in the attp40 landing site using the phiC31-based integration system. (B) Top: representative Western blots of antennal protein extracts from the indicated genotypes, probed with an anti-LUSH antibody (upper panel) or a control anti-IR8a antibody (lower panel). Genotypes (in this and all following figures): wild-type flies are *w^1118^*. *lush* ^−/−^ null mutant flies are *lush^1^/lush^1^*. Transgenic LUSH flies are *LUSH^x^/LUSH^x^;lush^1^/lush^1^*, where “*LUSH^x^*” refers to the indicated LUSH transgene. Bottom: relative quantification of LUSH expression in the indicated genotypes. The levels of IR8a were used as a protein loading control. The mean of the optical density (OD) arbitrary units (red bar) ± SD of four independent extracts for each genotype are shown. Statistical analysis (ANOVA and Dunnett's multiple comparison tests using the wild-type mean as control) showed a significant difference in LUSH expression between wild-type and *lush* ^−/−^ genotypes (**p<0.01) but no significant differences with the extracts from flies expressing the different LUSH transgenes. (C) Immunofluorescence with anti-LUSH (green) and anti-ORCO (magenta) antibodies on antennal cryosections of wild-type, *lush* ^−/−^ mutant, and transgenic LUSH animals. The scale bar represents 20 µm. A higher magnification detail of one optical section of the wild-type antennal section shows two ORCO-positive neurons flanked by LUSH-positive auxiliary cells. Similar restriction of LUSH expression to auxiliary cells was observed in all genotypes; overlap of green and magenta signals is due to auxiliary cells overlaying neurons in a different optical section.

To test the activity of in vivo-expressed mutant LUSH proteins, we first generated a wild-type genomic *lush* rescue construct, which spans the entire transcription unit and flanking intergenic sequences (Figure 2A). This construct, referred to hereafter as LUSH^wt^, is expected to contain all regulatory sequences necessary to recapitulate endogenous *lush* expression. By site-directed mutagenesis, we generated three additional *lush* constructs encoding proteins equivalent to the recombinantly expressed LUSH variants previously analyzed (LUSH^F121A^, LUSH^F121W^, and LUSH^D118A^) [29]. Each transgene was integrated in the same genomic location by phiC31-mediated germline transformation [32] and crossed into a *lush* null mutant background [33] to generate flies that are genetically identical except for the missense mutations within the transgenic *lush* coding sequence.

We first compared LUSH expression in these fly strains by Western blot analysis of antennal protein extracts using an anti-LUSH antibody. In all genotypes, we observed a single prominent band, which corresponds to LUSH as it co-migrates with endogenous LUSH in wild-type extracts and is absent in *lush* mutant extracts (Figure 2B). Relative quantification of LUSH levels showed that there is no statistical difference in the expression of the mutant or wild-type transgenic LUSH proteins when compared to endogenous LUSH (Figure 2B). There is some variability between extracts from the same genotype, which is probably due to the difficulty of reproducible protein extraction from the small, cuticular, antennal structures.

Immunofluorescence for LUSH and ORCO on antennal sections confirmed that all transgenic LUSH variants are expressed in auxiliary cells surrounding ORCO-positive OSNs (immunofluorescence detection of secreted LUSH in sensillar lymph is difficult, probably because this extracellular fluid is largely lost during tissue preparation and staining). These LUSH-expressing cells are located within the distal region of the antenna where pheromone-sensing trichoid sensilla are found (including those housing OR67d neurons), in a pattern indistinguishable from endogenous LUSH (Figure 2C).

### Mutation of LUSH^F121^ Does Not Affect the Sensitivity of OR67d Neurons to cVA

In cVA/LUSH complexes, the pheromone directly interacts with F121 in the C-terminal tail, suggesting that this residue has a central role in triggering the cVA-induced conformational change of LUSH [29]. It was hypothesized that substitution of F121 by a smaller residue (such as alanine) or a larger residue (such as tryptophan) might reduce or enhance, respectively, this conformational change. Indeed, recombinant LUSH^F121A^—infused through the recording electrode into OR67d sensilla (also referred to as T1 sensilla [29]) of *lush* mutants—restored sensitivity to cVA that is ∼50-fold lower than that conferred by recombinant wild-type LUSH across a 1,000-fold range of pheromone concentrations [29]. Conversely, infusion of recombinant LUSH^F121W^ conferred a 5-fold increase in sensitivity of OR67d neurons to cVA [29]. Together, these observations were consistent with the idea that cVA-induced conformational changes in LUSH underlie OR67d neuron activation.

**Figure 3:**
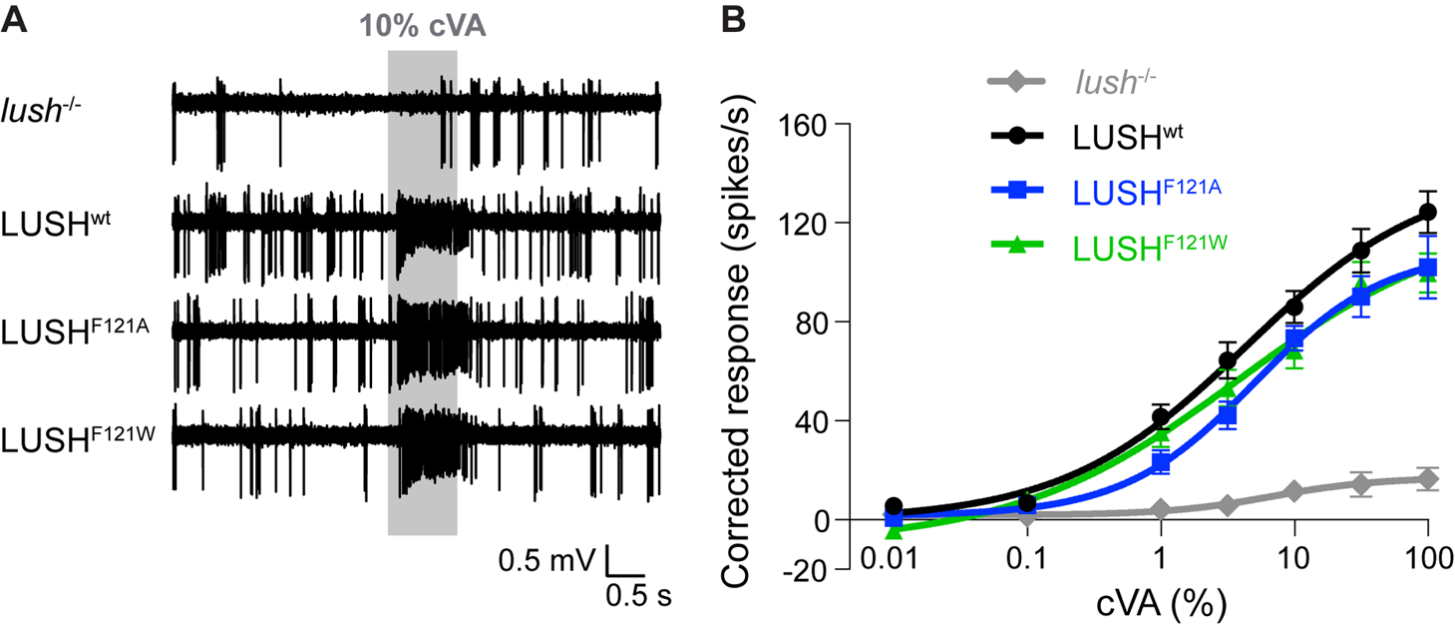
Mutations in LUSH^F121^ do not affect the sensitivity of OR67d neurons to cVA. (A) Representative traces of extracellular electrophysiological recordings of OR67d neurons in flies stimulated with 10% cVA in *lush* ^−/−^, LUSH^wt^, LUSH^F121A^, and LUSH^F121W^ flies (see Figure 2 legend for details of genotypes). The grey bar indicates the stimulus time (1 s). (B) Dose-response curves of OR67d neurons to cVA in the genotypes in (A). Mean responses are plotted (±SEM; *n* = 12–16 sensilla; ≤3 sensilla per animal). Curves were fitted using a log versus response-variable slope model with Prism-GraphPad software. There are no statistically significant differences in cVA sensitivity due to genotype between all the transgenic lines tested (ANOVA, *p = *0.1183). There is a slightly significant decrease in cVA-sensitivity at 100% cVA stimulation for LUSH^F121W^ when compared to LUSH^wt^ (*p*<0.05, Tukey's multiple comparison tests).

We tested whether these properties could be recapitulated by transgenic LUSH variants by measuring cVA-evoked responses in OR67d neurons in LUSH^wt^, LUSH^F121A^, and LUSH^F121W^ animals in single sensilla electrophysiological recordings. All three transgenes restore sensitivity to this pheromone in a *lush* mutant background (Figure 3A). However, in contrast to the observations with recombinant LUSH [29], neuronal responses across a 10,000-fold range of cVA concentrations were very similar across all three genotypes (Figure 3B). The only statistically significant difference between these strains was observed at the highest stimulus concentration, where LUSH^F121W^ flies exhibited slightly lower cVA sensitivity than LUSH^wt^ (Figure 3B). This result is contrary to the report that recombinant LUSH^F121W^ enhances cVA sensitivity [29].

### Mutation of LUSH^D118^ Does Not Increase Spontaneous Activity in OR67d Neurons

A second residue in LUSH implicated in cVA-evoked conformational activation is D118, which is predicted to form a salt bridge with K87 in apo LUSH that is disrupted upon cVA binding (Figure 1C) [29]. It was hypothesized that this salt bridge maintains LUSH in an “inactive” state, leading to the prediction that mutation of D118 would produce an “activated” form of LUSH. Consistently, recombinant LUSH^D118A^ was found to induce—in the absence of cVA—increased activity in OR67d neurons compared to recombinant wild-type LUSH, up to the level observed in wild-type OR67d neurons stimulated with 1% cVA [29]. This LUSH^D118A^-evoked activity depended upon both OR67d and SNMP [29].

**Figure 4:**
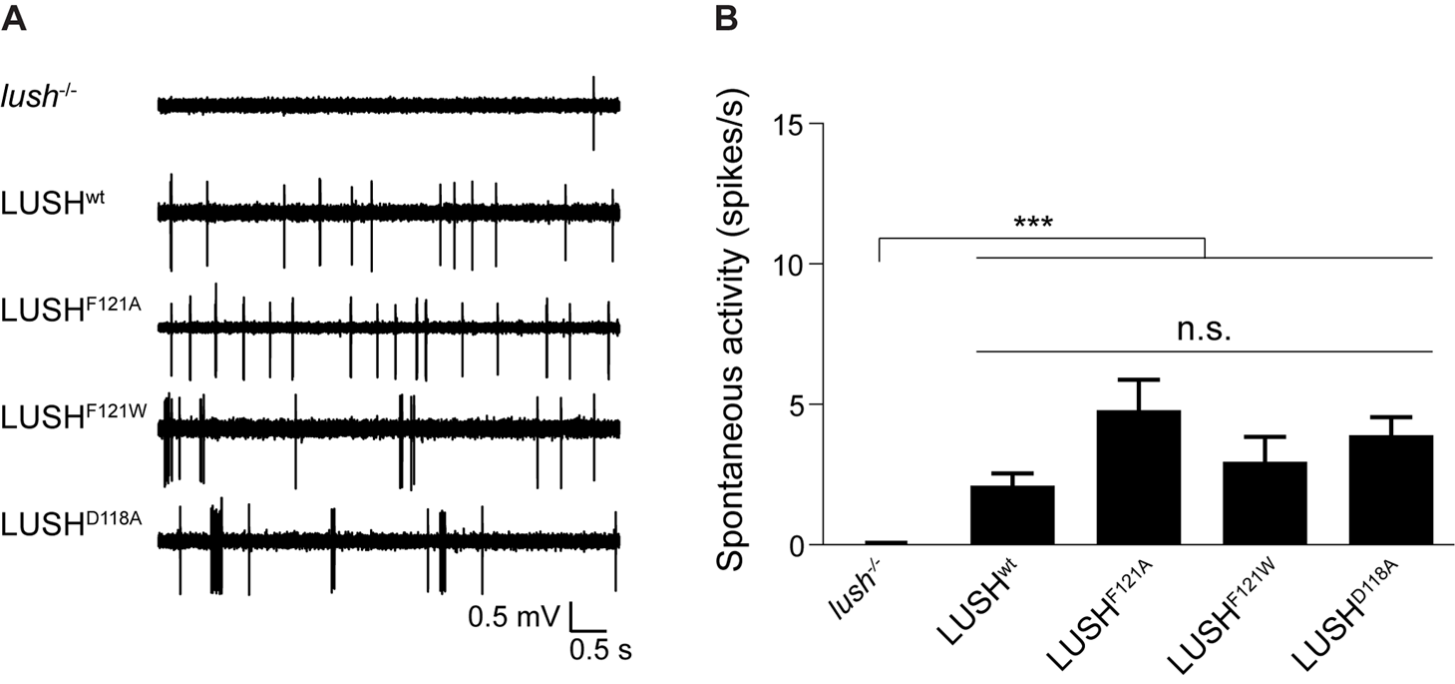
Mutation of LUSH^D118^ does not increase spontaneous activity in OR67d neurons. (A) Representative traces of spontaneous activity in OR67d neurons in *lush* ^−/−^, LUSH^wt^, LUSH^F121A^, LUSH^F121W^, and LUSH^D118A^ flies. (B) Quantification of mean spontaneous activity in the genotypes in (A) (±SEM; *n* = 19–20 sensilla; ≤3 sensilla per animal). There are no statistically significant differences in spontaneous activity due to genotype between all the transgenic lines tested (Kruskal-Wallis, *p = *0.0610). Only *lush* ^−/−^ null mutants have a statistically significant decrease in spontaneous activity when compared to all the other genotypes (*p*<0.0001, Dunn's multiple comparison tests).

To test whether D118 is critical in transgenically expressed LUSH, we measured spontaneous activity in OR67d neurons in LUSH^wt^ and LUSH^D118A^ flies. We also tested the LUSH^F121A^ and LUSH^F121W^ lines, because recombinant LUSH^F121A^ was unable to rescue the loss of spontaneous activity in OR67d neurons observed in *lush* mutants thereby providing a second piece of evidence linking LUSH conformational changes and OR67d neuronal firing [24],[29]. We observed that all LUSH transgenes restored spontaneous firing of OR67d neurons (Figure 4A). Quantification of this activity revealed minor variation in their mean firing frequencies (∼1–3 spikes/s), but these differences were not statistically significant across genotypes (Figure 4B). Importantly, LUSH^D118A^ flies did not exhibit the elevated level of spontaneous activity reported for recombinant LUSH^D118A^. Surprisingly, spontaneous firing was also observed in the LUSH^F121A^ genotype (Figure 4B), which is inconsistent with the activity of recombinant LUSH^F121A^ but consistent with our observation that the transgenic protein supports cVA-evoked activity as effectively as LUSH^wt^ (Figure 3B).

The loss of basal activity in OR67d neurons in the absence of LUSH provided an initial hint for a direct role for this OBP in promoting OR67d activity [24]. However, while spontaneous activity is highly reduced in *lush* mutants, it is not completely abolished (0.05±0.01 spikes/s mean ± standard error of the mean (SEM); *n* = 19) (Figure 4A and 4B). Moreover, OR67d/ORCO evokes robust spontaneous activity in the absence of LUSH when mis-expressed [14],[21] and in OR67d sensilla lacking both LUSH and SNMP [21]. The origin of this *lush* phenotype and its relevance for cVA signal transduction therefore remains unclear. One possible explanation is that loss of LUSH from the sensillum lymph changes the physiological properties of this medium with indirect effects upon OR67d neuron excitability.

### Mutation of LUSH^D118^ Does Not Affect the Responses of OR67d Neurons to cVA

**Figure 5:**
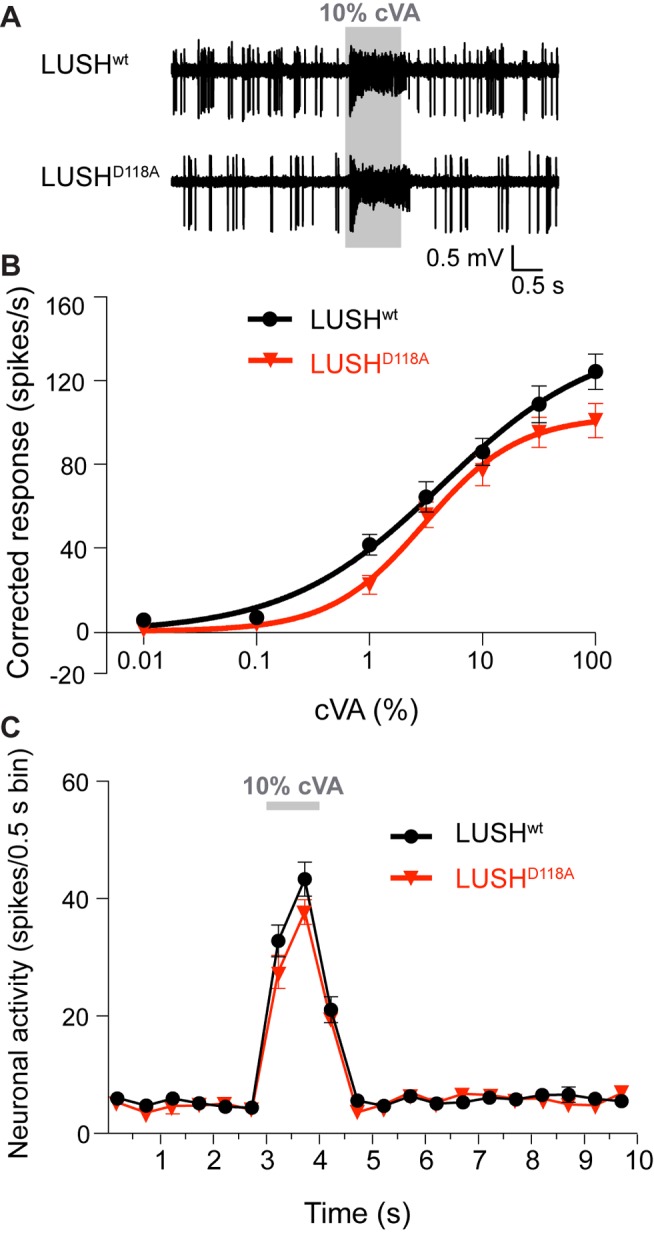
Mutation of LUSH^D118^ does not affect the responses of OR67d neurons to cVA. (A) Representative traces of extracellular electrophysiological recordings of OR67d neurons in LUSH^wt^ and LUSH^D118A^ flies stimulated with 10% cVA. The grey bar indicates the stimulus time (1 s). (B) Dose-response curves of OR67d neurons to cVA in the genotypes in (A). Mean responses are plotted (±SEM; *n* = 15–16 sensilla; ≤3 sensilla per animal). The trace and quantified data for LUSH^wt^ are the same as in Figure 3. There are no statistically significant differences in cVA sensitivity due to genotype (ANOVA, *p* = 0.1183). There is a small but significant decrease in cVA-sensitivity at 100% cVA stimulation for LUSH^D118A^ when compared to LUSH^wt^ (*p*<0.05; Tukey's multiple comparison tests). (C) Peristimulus time histograms of cVA-evoked responses in OR67d neurons in LUSH^wt^ and LUSH^D118A^ flies. There are no statistically significant differences in neuronal responses due to genotype (ANOVA, *p* = 0.3553). There is a small but significant decrease in cVA sensitivity in LUSH^D118A^ compared to LUSH^wt^ flies only in the 3.5–4-s time bin (*p*<0.05; Sidak's post hoc multiple comparison tests).

One important difference between the experiments with recombinant LUSH^D118A^ [29] and transgenic LUSH^D118A^ (Figure 4) is that the former protein was provided acutely to sensilla during electrophysiological recordings, while the latter is present continuously in the lymph. We considered the possibility that transgenic LUSH^D118A^ is also constitutively active but that its constant presence leads to desensitization of OR67d neurons. If this were the case, we would not expect these OSNs to be able to respond to cVA. However, we observed that LUSH^D118A^ transgenic flies still display robust responses to a 10,000-fold range of cVA concentrations (Figure 5A and 5B). These responses are statistically indistinguishable from LUSH^wt^, except at the highest dose presented (Figure 5B). Analysis of the temporal dynamics of cVA-evoked neuronal activity revealed that both the onset and the decay of cVA responses are very similar for LUSH^D118A^ and LUSH^wt^ (Figure 5C).

These observations indicate that the transgenic expression of LUSH^D118A^ does not lead to desensitization of OR67d neurons. Rather, they highlight an important functional difference between recombinant and transgenic LUSH^D118A^: recombinant LUSH^D118A^ was reported to be unable to support further activation of OR67d neurons when cVA is presented [29]. This observation was suggested to reflect the fact that the “conformationally activated” LUSH^D118A^ could not be further activated by cVA. By contrast, our results indicate that transgenic LUSH^D118A^ supports cVA-evoked activity essentially identically to wild-type LUSH. Because of these differences, we analyzed an independently generated LUSH^D118A^ transgenic line (Figure S1) [34]. Spontaneous activity in OR67d neurons in these flies was not elevated (indeed, it was slightly lower) compared to a control wild-type rescue transgenic strain (Figure S1A and S1B), and cVA-evoked responses were essentially normal (Figure S1C and S1D), consistent with the properties of our LUSH^D118A^ transgene.

### Re-examination of the Structural Dynamics of LUSH

Given the discrepancies between the properties of transgenic and recombinant LUSH proteins, we re-examined the published LUSH structures to determine the relationship between the presence and type of ligand and the conformation of this OBP (Table 1). We first superposed the 12 available structures of LUSH (Figure S2A) [25]–[27],[29]. Within each asymmetric unit in LUSH crystals (except one: apo LUSH, Protein Data Bank [PDB] ID 1OOI), there are two protein molecules, referred to as monomer A and B in cVA/LUSH [29] (dimerization of other OBPs has been observed in crystal structures [30], and there is no evidence that this reflects an in vivo biochemical property of LUSH). All non-cVA bound LUSH structures (except butanol/LUSH^T57A^ PDB 3B87) possess similar conformations of loop 116–122 (Figure S2A), and the cVA/LUSH complex is distinct from these structures within this region. However, at least three cVA/LUSH conformations have been observed in the crystal: monomer A of cVA/LUSH exists in two different conformations, and the structure of monomer B is distinct from either of these (Figure S2A) [29]. Thus, interaction of cVA with LUSH, as captured by X-ray crystallography, reveals substantial conformational heterogeneity rather than a single “activated” form of this complex that was postulated to be recognized by pheromone receptors [29].

**Table 1:** Conformational heterogeneity of LUSH crystal structures.

Structure	Apo LUSH	Apo LUSH	cVA LUSH	Apo LUSH^D118A^	Ethanol LUSH	Propanol LUSH	Butanol LUSH	Butanol LUSH^S52A^	Ethanol LUSH^T57A^	Butanol LUSH^T57A^	Ethanol LUSH^S52A^	Ethanol LUSH^T57S^
PDB ID	1OOI	1T14	2GTE	2QDI	1OOF	1OOG	1OOH	3B6X	3B88	3B87	3B7A	3B86
Space group	P6_1_	P4_3_	P2_1_2_1_2_1_	P4_3_	P4_3_	P4_3_	P4_3_	P4_3_	C222_1_	P2_1_2_1_2_1_	P4_3_	P4_3_
Resolution (Å)	2.04	1.86	1.40	2.0	1.49	1.45	1.25	2.00	2.00	2.00	1.90	2.00
pH	6.5	4.6	8.5	4.0	4.6	4.6	4.6	4.6	—	—	4.6	—
Distance (Å): Oδ1/2 (D118 A) −Nζ (K87 A)	5.33	3.55/4.54	4.91/7.70	—	3.75/4.54	3.33/4.48	2.81/4.46	2.62	3.43	4.44	4.26	4.95
Distance (Å): Oδ1/2 (D118 B) −Nζ (K87 B)	—	4.42	8.09	—	3.03	4.41	2.70	4.71	4.63	5.70	3.57	2.92

Comparison of different LUSH structures belonging to four different space groups and five crystal forms reported in [25]–[27],[29]. All structures except 1OOI comprise two monomers in the asymmetric unit. The distances between K87 Nε and K118 Oδ1 or Oδ2 (whichever is closest) were measured with Coot [58]. The distances separated by a slash are those measured for side chains with alternate conformations.

We re-assessed the possible presence of a salt bridge between D118 and K87 in all LUSH structures by measuring minimal distances between the D118 anionic carboxylate group (Oδ1/Oδ2) and the K87 cationic ammonium K87 (Nζ) (Table 1); this value must be less than 4 Å if such an ionic bond forms [35]. In the cVA/LUSH complex (PDB 2GTE), D118 and K87 are sufficiently distant in any conformation to be unable to form a salt bridge (Table 1), as described [29]. However, this salt bridge is not a consistent feature of the structures of apo or alcohol-bound LUSH (Table 1): in two cases (PDB 1OOI and 3B87), no salt bridge is observed in either monomer in the asymmetric unit, while in eight other cases, the salt bridge is established reliably in only one monomer. These observations are inconsistent with the idea that a D118-K87 salt bridge maintains LUSH in an inactive state and that cVA binding specifically disrupts this bond to activate LUSH [29].

### Requirement for LUSH Can Be Bypassed by High Concentrations of cVA

**Figure 6:**
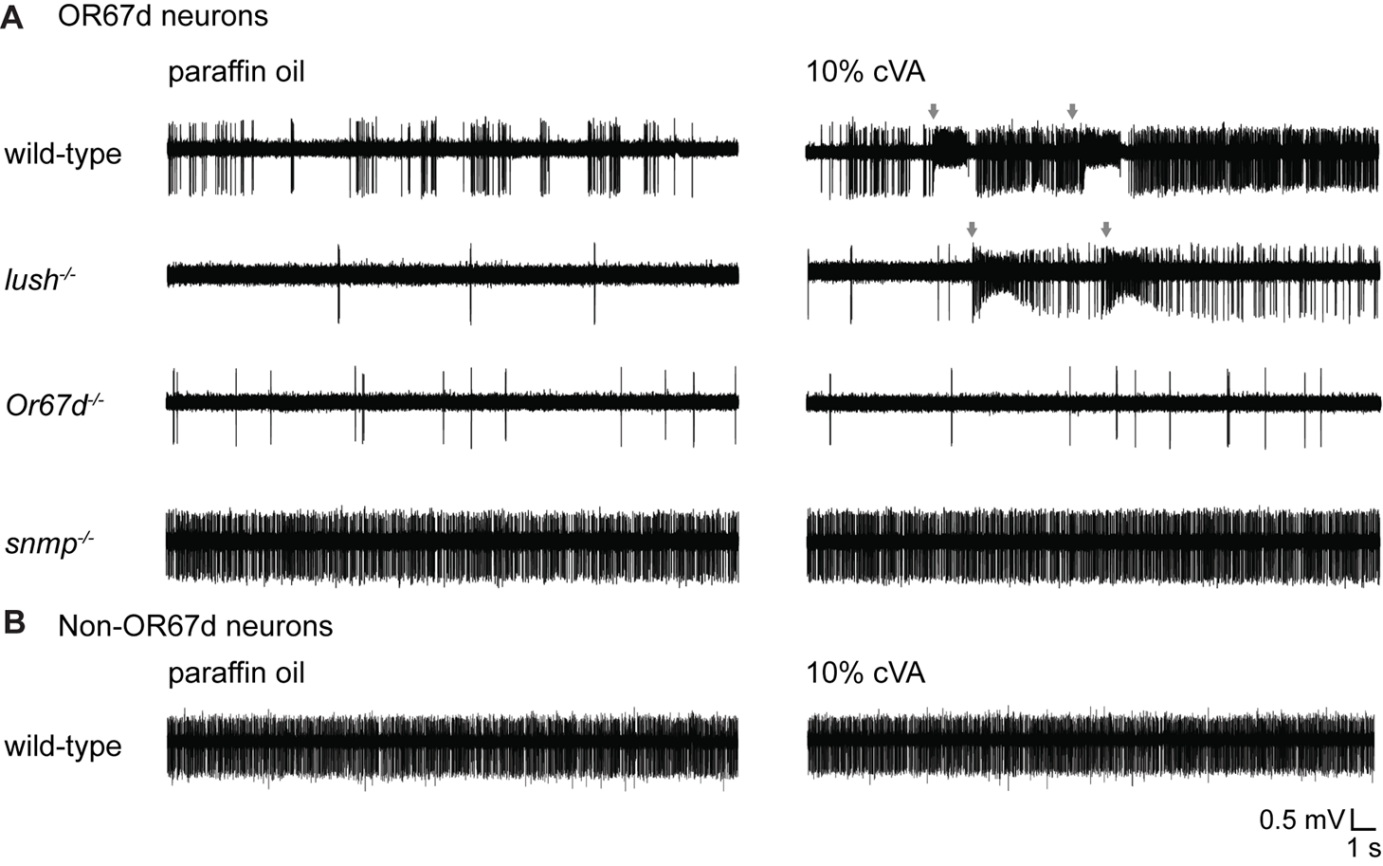
Requirement for LUSH can be bypassed by high concentrations of cVA. (A) Representative traces of extracellular electrophysiological recordings of OR67d neurons in flies in a close-range stimulation assay for wild-type, *lush* ^−/−^, *Or67d* ^−/−^ (*Or67d^GAL4^/Or67d^GAL4^)*, and *snmp* ^−/−^ (*snmp^1^/snmp^1^*) flies. During the 23 s recordings shown, a strip of filter paper spotted with paraffin oil (left traces) or 10% cVA (right traces) was moved, using a manual micromanipulator, within ∼0.1 mm of the antenna twice for ∼1 s, separated by a ∼4–5 s interval. The grey arrows indicate the approximate time of close approach of the cVA stimulus in wild-type and *lush* ^−/−^ mutant sensilla. In other genotypes cVA did not evoke a response, and paraffin oil did not evoke a response in any genotype, so the precise timing of stimulation could not be determined from these traces. Additional examples of traces are provided in Figure S3. (B) Representative traces of extracellular electrophysiological recordings of non-OR67d neurons in a wild-type fly stimulated with paraffin oil (left traces) or 10% cVA (right traces) in a close-range approach assay as in (A).

The model that the cVA/LUSH complex directly activates OR67d neurons predicts that LUSH should be essential for cVA-evoked activation of this receptor. However, previous studies demonstrated that OR67d (with or without SNMP) can respond weakly to cVA in the absence of LUSH when mis-expressed in a neuron normally insensitive to this pheromone [14],[21],[29]. As heterologous receptor expression may not necessarily reflect activity of the endogenous signal transduction pathway, we examined the responses of OR67d neurons to cVA in the presence or absence of LUSH by adapting a close-range stimulation assay [14]. Typically, odors are presented to antennae by delivering (and diluting) in an airstream the headspace of a filter paper impregnated with the stimulus odor [36]. In our close-range assay, we presented cVA by approaching the antenna (within ∼0.1 mm) with a filter paper strip on which 2 µl of a 10% solution of cVA (or the pure paraffin oil solvent) was spotted. Contact between the filter paper and antenna was completely avoided. For each sensillum tested, we approached twice (for ∼1 s each, separated by a ∼4–5 s interval) with a filter paper containing paraffin oil and twice with a filter paper containing cVA. Wild-type OR67d sensilla responded consistently and strongly to presentation of cVA (24/24 approaches; mean corrected responses upon first stimulus approaches ± SEM = 112.2±8.4 spikes/s, *n* = 12) (Figure 6A). No responses were observed to paraffin oil indicating that there is no mechanosensory artifact due to close approach of the filter paper. In *lush* mutants, we also observed robust, albeit lower, responses to presentation of cVA but not paraffin oil (24/24 approaches; mean corrected responses upon first stimulus approaches ± SEM = 60.2±6.7 spikes/s, *n* = 12) (Figures 6A and S3). The existence of these cVA-evoked responses is consistent with observations that delivery of cVA in an airstream can evoke weak activity in OR67d neurons in *lush* mutant flies at the highest stimulus concentrations (Figure 3B and [29]) and suggests that our close-approach assay merely achieves higher stimulus concentrations than those possible by airstream delivery. These observations indicate that while LUSH is important for high sensitivity responses to cVA, it is not essential for cVA-evoked activity in OR67d neurons.

To demonstrate that LUSH-independent activation of OR67d neurons by cVA is specific and not an artifact of high stimulus concentrations, we also tested mutant flies lacking the neuronal receptors OR67d or SNMP in our close-range assay. Like *lush* mutants, *Or67d* mutant sensilla exhibit low spontaneous firing frequency, but no responses to cVA were observed (0/20 approaches) (Figures 6A and S3). As observed previously [20],[21], *snmp* mutant flies display—for unclear reasons—elevated spontaneous firing of OR67d neurons, but these sensilla also failed to exhibit evoked activity upon close-range stimulation by cVA (0/24 approaches) (Figures 6A and S3). Together, these results confirm that both neuronal membrane receptors are, in contrast to LUSH, essential for cVA-evoked activity in endogenous OR67d neurons [20]–[23]. Moreover, they indicate that the responses observed in *lush* mutant sensilla are unlikely to be due to non-specific activation of these neurons by high concentrations of pheromone.

We further verified that close-range stimulation of OR67d neurons by cVA is specific by recording responses in non-OR67d trichoid sensilla. These sensilla can be easily distinguished from those housing OR67d OSNs because they contain two or three different neurons that exhibit multiple distinct spike amplitudes and have higher basal activity levels than OR67d OSNs [24]. While 20/20 OR67d sensilla respond to close approach with cVA, only 1/20 non-OR67d sensilla displayed a detectable response (Figures 6B and S3). The sole responding sensillum could potentially house an OR65a neuron, which is weakly sensitive to cVA [14].

## Discussion

We have tested a model proposing that the *Drosophila* pheromone cVA is detected through conformational activation of the OBP LUSH, which then acts as the ligand for pheromone-sensing neurons [29]. This model was based upon analysis of the structural and functional properties of several site-directed mutant LUSH proteins that were expressed recombinantly and delivered acutely to cVA-sensing neurons. In this work, using a transgenic-expression system for these LUSH mutant proteins, we have made several observations that are inconsistent with this model. We discuss the basis for these discrepancies and re-consider the role of LUSH in pheromone sensing.

### Functional Differences of Recombinant and Transgenically Expressed LUSH

The discrepancy between our findings and those previously reported [29] are likely to reflect the difference in how wild-type and mutant LUSH proteins are provided to cVA-sensing sensilla. Recombinant LUSH proteins were expressed (lacking the N-terminal signal peptide) in *Escherichia coli*, and purified and re-folded from inclusion bodies [24]–[26],[29]. These proteins were then introduced into individual sensilla via a glass electrode, which was simultaneously used for extracellular recording of action potentials of the OR67d neurons [24],[29]. It is presumed that LUSH passes from the electrode to the sensillar lymph by diffusion, although the precise diffusion rate and the final concentration of LUSH in the lymph at equilibrium are difficult to determine. Nevertheless, this method provides a way to assess the effect of acute (i.e., within a timescale of 5–30 min) delivery of LUSH on spontaneous and cVA-evoked activity [24],[29].

We provided LUSH via a transgenic genomic rescue construct that appears to contain all necessary transcriptional and translational regulatory sequences to recapitulate endogenous LUSH expression. Importantly, we have compared the function of mutant LUSH transgenes with a wild-type transgene inserted at the same genomic location; these flies are genetically identical except for the desired single amino acid substitutions in LUSH. As transgenically expressed LUSH is supplied both during development and in the mature olfactory system, it is conceivable that tonic exposure of OR67d neurons to mutant versions of LUSH changes their activity. This could account for the higher basal activity observed in OR67d neurons after acute delivery of the presumed constitutively active LUSH^D118A^ compared to the effect of the same protein expressed transgenically [29],[34]. We believe that this explanation is unlikely, however, as transgenic LUSH^D118A^ fully supports cVA-evoked activity, indicating that OR67d neurons have not adapted to tonic exposure to this mutant protein. Indeed, our finding that LUSH^D118A^ rescues the ability of OR67d neurons to respond to cVA conflicts with the previous demonstration that recombinant LUSH^D118A^ does not confer cVA sensitivity [29].

We argue that the transgenically expressed LUSH is more likely to reflect the activity of the wild-type and mutant variants than when provided recombinantly. Like other OBPs, LUSH is expressed in auxiliary cells that flank OSNs and must pass through their endomembrane system before being secreted into the sensillar lymph [37],[38]. During this transit, LUSH presumably forms (like other OBPs) intramolecular disulfide bridges in the lumen of the endoplasmic reticulum; it is unknown whether the protein undergoes other post-translational modifications. The transgenic approach allows stable expression of LUSH proteins that are likely to follow this same transport pathway, and avoids the technical challenges of delivering purified, recombinant LUSH.

### The Roles of LUSH in Chemosensation

Several functions of LUSH have been described since the identification of this OBP gene in an enhancer-trap screen [33]. *lush* mutants were initially shown to display behavioral defects in avoidance of high concentrations of short-chain *n*-alcohols [33]. Subsequently, biochemical and structural studies demonstrated that LUSH binds alcohols, with encapsulation of single alcohol molecules in the binding pocket serving to conformationally stabilize LUSH [25],[26]. Although another study suggested that LUSH binds aromatic compounds and not alcohols in vitro [28], electrophysiological analysis showed that *lush* mutants display defects in alcohol-evoked inhibition of neural activity in certain classes of (non-cVA sensing) trichoid OSNs [24]. However, the identity of these alcohol-sensitive neurons, the role of LUSH in regulating their activity, and how this relates to the alcohol-avoidance defects of *lush* mutants remain unknown.

The dramatic reduction in cVA-sensitivity in OR67d neurons in *lush* mutants provided the first evidence for a role for this OBP in olfactory transduction in vivo. LUSH is highly expressed in most, if not all, trichoid sensilla in the antenna [39], which house nine OSN classes expressing different ORs [40]. Two of these receptors, OR67d and OR65a, respond to cVA [14] but the ligands for the remaining ORs are unknown. The presence of LUSH in the lymph fluid surrounding these receptors opens the possibility that this OBP—like SNMP [20],[21]—acts in the detection of multiple pheromones. Indirect evidence for this possibility comes from consideration of the behavioral phenotype of *lush* mutants. Loss of LUSH was initially linked to defects in cVA-induced aggregation behavior but not sexual behaviors [24]. More recently, cVA (or artificial) stimulation of OR67d neurons has been shown to be necessary and sufficient both to inhibit male courtship behavior and to promote female receptivity [22],[34]. The absence of reported courtship defects in *lush* mutants could be explained, for example, if LUSH was involved in recognition of other pheromones by different ORs with opposite functions in regulating sexual behavior. While cVA is the only volatile pheromone identified so far in *Drosophila*, LUSH can bind other insect pheromones, suggesting its ligand-binding pocket can accommodate structurally diverse, long-chain hydrocarbons [41].

In sum, evidence from behavioral, biochemical, structural, and expression studies suggests that LUSH plays several roles in chemosensation, with a variety of odor ligands and different ORs. According to the proposed model [29], the unique ability of a cVA/LUSH complex to activate OR67d neurons is a reflection of the distinct conformation of the complex. However, our re-analysis of the available X-ray crystal structures argues that current data for LUSH do not unambiguously support this possibility for two reasons. First, as previously noted [29], cVA/LUSH complexes are structurally heterogeneous, and it remains unclear which—if any—structure reflects the in vivo conformation of cVA/LUSH. Second, the presumed D118-K87 salt bridge that was suggested to maintain LUSH in an “inactive” state in OR67d sensilla [29] is not a consistent feature of either apo LUSH or complexes of this OBP with non-pheromonal ligands. Indeed, because a LUSH^D118A^ crystal structure was reported to resemble the cVA/LUSH structure [29], our demonstration that LUSH^D118A^ behaves similarly to wild-type LUSH in vivo questions the functional significance of the observed conformational changes. It is possible that mutagenesis of other (or additional) residues within LUSH may more precisely mimic the pheromone-bound state(s), but it is not obvious which residues should be targeted. It also remains conceivable that cVA binding induces structural alterations in LUSH that are not revealed by X-ray crystallography but which are relevant for pheromone signaling; investigation of this possibility would require new ways of visualizing the interaction of cVA with LUSH.

### Mechanism of LUSH in cVA Detection

Our genetic, electrophysiological, and structural analyses of LUSH fail to find evidence in support of the conformational activation model [29], raising the question of how LUSH participates in cVA detection. The close-approach stimulation assay reveals that cVA can activate OR67d neurons in the absence of LUSH. While the quantity of pheromone entering the sensilla in this assay is likely to be in excess of that encountered in nature [29], our control experiments show that this stimulation is specific to OR67d neurons and depends upon the neuronal receptor components SNMP and OR67d. Moreover, the LUSH-independent stimulation of OR67d is in accord with several previous analyses of this receptor [14],[21],[29]. Together, these observations argue for a mechanism in which cVA must directly activate OR67d, and indicate that the specificity of pheromone detection resides principally in this pheromone ligand/OR interaction. This proposition is inline with observations that chemically diverse hydrocarbon pheromones in other insects can, with varying degrees of efficiency, activate their cognate ORs in the absence of OBPs in several different in vivo and in vitro heterologous expression systems [15],[22],[42]–[48].

The major loss of sensitivity to cVA in *lush* mutants [24] indicates an important role for this OBP in pheromone detection, and likely an essential one at ecologically relevant pheromone concentrations. While our experiments do not address how LUSH functions mechanistically, we suggest that encapsulation of cVA by LUSH helps first to solubilize the hydrophobic pheromone molecules in the aqueous sensillar lymph and to protect them from degradation by ODEs [49], but that this OBP must ultimately deliver and release cVA to the neuronal pheromone receptors. The combined effect of these actions of LUSH would be to produce a concentration of pheromone available to bind OR67d that is several orders of magnitude greater than would be achieved without this OBP. These ideas are consistent with the in vivo analysis of cVA detection in wild-type and *lush* mutants ([24],[29] and this work), but also derive from studies on pheromone-binding OBPs in heterologous systems. For example, several moth pheromone receptors, when expressed in cultured mammalian cells, can respond to high concentrations of their cognate ligand solubilized in dimethyl sulfoxide but that provision of the pheromone together with an OBP/PBP negates the requirement for this organic solvent [45],[46],[50]. How LUSH might release cVA to the pheromone receptors is unclear, but would presumably require a reversal of the cVA-evoked conformational change. In other OBPs/PBPs, several lines of evidence support a pH-dependent conformational regulation that controls ligand release, at least in vitro (reviewed in [11]). We also suggest that binding of LUSH with one or more of the neuronal membrane receptors (SNMP, OR67d, and/or ORCO) could trigger cVA release.

OBPs were first identified over 30 years ago [51]. The size of these repertoires in insect genomes and the diversity in their sequences, expression patterns, and in vitro biochemical properties argue for a widespread role in chemical detection in insects [11],[30],[52],[53]. To date, the loss-of-function genetic analysis of LUSH [24] is the most compelling demonstration of the importance of this role. Defining the precise mechanism by which LUSH and other OBPs act in vivo, however, still awaits.

## Materials and Methods

### *Drosophila* Genetics

*Drosophila* stocks were maintained on conventional food medium under a 12 h light∶12 h dark cycle at 25°C. The wild-type genotype (Figures 2, 6, and S3) was *w^1118^*. Previously described mutant alleles and transgenic lines used were *lush^1^* [33], *Or67d^GAL4^* [22], *snmp^1^* [21], a *lush* rescue transgene (referred to here as *lush^wt-DS^*) [33], and a *lush^D118A^* transgene (referred to here as *lush^D118A-DS^*) [34] (kindly provided by Dean Smith, UT-Southwestern). New transgenic lines were generated with the phiC31-based integration system [32], using the attP40 landing site [54], by Genetic Services Inc. The identity of *lush* transgenic flies was re-verified by PCR amplification of the transgenic *lush* coding sequence and sequencing.

### Molecular Biology

Gene-specific primers were designed using Primer3 (http://frodo.wi.mit.edu/) to amplify a genomic region of 3,228 bps, starting 958 bp upstream of the *lush* start codon to 1,693 bp downstream of the *lush* stop codon; this region includes the entire *lush* transcription unit and flanking intergenic non-protein coding sequences. The wild-type *lush* transgene (LUSH^wt^) was generated by PCR amplification with these primers on genomic DNA prepared from the genome-sequenced strain [55], using the KAPA HiFi PCR kit (Kapa Biosystems); the resulting PCR product was T:A cloned into pGEM-T Easy (Promega), sequenced, and subcloned with restriction enzymes (*XhoI*/*XbaI*), whose recognition sites were incorporated into the PCR primers, into the pattB vector [32]. Point mutations in the *lush* coding sequence were introduced by site-directed mutagenesis of pGEM-T *lush^wt^*, which was fully sequence-verified both before and after subcloning into pattB.

### Histology

Immunofluorescence on antennal cryosections was performed as described [56].

#### Primary antibodies

Rabbit polyclonal antibodies against LUSH were raised against the synthetic peptide KFKLKTEDLDRLRVGDFN, and guinea pig polyclonal antibodies against ORCO were raised against the synthetic peptide SSIPVEIPRLPIKS. Both were generated and affinity purified by Proteintech Group, Inc and used at 1∶1,000 (ORCO) or 1∶2,000 (LUSH).

#### Secondary antibodies

Alexa488- and Cy3-conjugated anti-rabbit IgG or anti-guinea pig IgG (Molecular Probes; Jackson Immunoresearch) were used at 1∶1,000. All microscopy was performed using a Zeiss LSM 510 Meta Upright Laser Scanning Confocal microscope. Confocal images were processed with ImageJ (Rasband, WS, ImageJ, US National Institutes of Health, Bethesda, Maryland, http://imagej.nih.gov/ij/, 1997–2012) and Adobe Photoshop CS4.

### Biochemistry

Approximately 200 third antennal segments were harvested by snap-freezing flies in a mini-sieve (Scienceware, Bel-Art Products) with liquid nitrogen and gently shaking them to break off and collect the appendages in a Petri dish under the sieve containing 0.1% Triton X-100. Antennal segments were selected under a binocular microscope and transferred with a pipette into an Eppendorf tube. After centrifugation at 12,000 *g* for 2 min, the liquid phase was removed, and the antennae disrupted using a TissueLyzer (Qiagen). Protein extracts were made by incubating lyzed antennae in 150 µl of extraction buffer (20 mM Tris [pH 7.5], 100 mM NaCl, 5 mM KCL, 1.5 mM MgCl_2_, 4% glycerol, 0.02% n-dodecyl-D-maltoside) for 1 h at 4°C, followed by centrifugation at 12,000 *g* for 15 min at 4°C. 30 µl of extract were separated on 4%–20% precast gels (NuSep) and transferred to Hybond-ECl membrane (Amersham), which were then probed with primary antibodies against LUSH (described above; diluted 1∶4,000) or IR8a (diluted 1∶5,000) [56]. Goat anti-IgG rabbit (Promega) or donkey anti-IgG guinea pig (Fitzgerald Industries International) secondary antibodies coupled to HRP were used to detect LUSH and IR8a proteins, respectively. Blots were developed with medical X-RAY films (Fujifilm) using the ECL Plus Western blotting detection system (GE Healthcare). The resulting films were scanned without any automatic gain and bands were quantified in ImageJ. A rectangular box with a width slightly smaller than the narrowest band was defined on the first lane of the image and then used to measure densities of all lanes from the same film. The background was calculated for each lane and subtracted from the density of the corresponding bands as described [57].

### Electrophysiology

Extracellular recordings of OSN activity in individual sensilla of 4- to 8-day-old male flies were performed as described [21],[36].

#### Spontaneous activity measurement

Spontaneous activity was quantified by counting the spikes in a 13 s window without stimulus, and dividing by 13 to obtain spikes/s. All measurements of basal neuronal firing were made in the complete absence of cVA, to avoid the residual presence of this pheromone in the close environment of the fly, which can artificially elevate basal firing frequency.

#### Odor presentation in an airstream

10 µl of odorant were added to a 6×7.5 mm absorbent strip (Sugi, Kettenbach), which was placed inside a 1 ml tuberculin syringe (Becton, Dickinson and Company). A charcoal-filtered airflow (35 ml/s) was used to deliver odors to the preparation through a 10 ml serological pipette that was trimmed to remove the tapered tip, and the cut end positioned 15 mm away from the preparation. Half of this airflow was diverted through the odor syringe during odor stimulation periods (1 s) under the control of the Syntech CS-55 Stimulus controller. cVA (Pherobank; purity 99%) was diluted v/v in paraffin oil as indicated in the figures. We found that the onset of cVA-evoked responses varied slightly (usually 200 ms) between animals of the same genotype recorded on different days, most probably owing to small variations in the position of the odor delivery apparatus relative to the preparation. For quantification of responses, we therefore determined the time of onset of the response of a control wild-type sensillum to 100% cVA for each recording session. Corrected responses for all recordings in the same session were quantified by counting spikes in a 0.5 s window from this time point, subtracting the number of spontaneous spikes in a 0.5 s window before stimulation, and doubling the result to obtain spikes/s. The only exception was made for *lush* mutant flies in which cVA-evoked responses are delayed approximately 800–1,000 ms. In this case, the corrected responses were quantified by counting spikes in a 0.5 s window from the response onset time point, subtracting the number of spontaneous spikes in a 0.5 s window before the response onset, and doubling the result to obtain spikes/s. Peristimulus time histograms (PSTHs) were generated by counting the numbers of spikes in 0.5 s bins from 3 s before to 7 s after stimulation for each trial. These values were then averaged across all trials.

#### Odor presentation at close-range

2 µl of odorant were added to the tip of a 1 mm filter paper (Whatman). Using a fine micromanipulator the filter paper tip was approached within ∼0.1 mm of the third antennal segment, without contacting it. The stimulus was presented twice in a recording window of 23 s. For those genotypes that showed cVA-evoked responses, the response was quantified in the first stimulus presentation as described above.

#### Statistical analyses

After performing a normality test on the data, we compared all genotypes for a given experiment by ANOVA or non-parametric ANOVA, with genotype as the main effect, and adjusted the alpha level for planned post hoc means comparisons.

## Supporting Information

Figure S1 **Functional analysis of an independent LUSH^D118A^ transgene (related to** **Figures 4** **and** **5** **).** (A) Representative traces of spontaneous activity in OR67d neurons in wild-type, *lush* ^−/−^, LUSH^wt-DS^, and LUSH^D118A-DS^ flies. (B) Quantification of mean spontaneous activity in the genotypes in (A) (±SEM; *n* = 19–23 sensilla; ≤3 sensilla per animal). Although we confirmed the ∼2-fold higher spontaneous activity in LUSH^D118A-DS^ flies compared to a wild-type control, as reported [34], the firing frequency is lower than that observed in a control transgenic LUSH^wt-DS^ strain, and falls within the range of spontaneous firing frequencies observed across our new transgenic LUSH lines (Figure 4B). (C) Representative traces of extracellular electrophysiological recordings of OR67d neurons in flies stimulated with 10% cVA in the genotypes in (A). The grey bar indicates the stimulus time (1 s). (D) Dose-response curves of OR67d neurons to cVA in the genotypes in (A). Mean responses are plotted (±SEM; *n* = 12–17 sensilla; ≤3 sensilla per animal). There are no statistically significant differences in cVA sensitivity due to genotype between wild-type, LUSH^wt-DS^, and LUSH^D118A-DS^ animals (ANOVA, *p = *0.3911).
(TIF)

Figure S2 **Conformational heterogeneity of LUSH crystal structures (related to** **Table 1** **).** (A) Ribbon view of the superimposed backbones of apo LUSH, various alcohol/LUSH complexes (grey), and three conformations of the cVA/LUSH complex (green, monomer A, conformation A; beige, monomer A, conformation B; violet, monomer B). The ligand, cVA, is depicted in stick form and colored green in the monomer A structure and violet in the monomer B structure. Ct, C-terminus. (B) Close-up of the regions corresponding to residues 83–87 and 115–123 of the structures shown in (A). The side chains of K87 (nitrogen atoms colored blue), D118 (oxygen atoms colored red), and F121 are represented in stick form. Note the large diversity of side-chain conformations. (C) Close-up of residues 83–87 and 115–123 for apo LUSH (grey) and the three conformations of cVA/LUSH, colored as in (A). The side chains of K87, D118, and F121 are represented by sticks. The cVA ligand is shown in yellow (monomer A) or pale violet (monomer B).
(TIF)

Figure S3 **Requirement for LUSH can be bypassed by high concentrations of cVA (related to** **Figure 6** **).** Additional traces of extracellular electrophysiological recordings of OR67d or non-OR67d neurons in the indicated genotypes stimulated in a close-range stimulation assay. During the 23 s recordings shown, a strip of filter paper spotted with paraffin oil (left traces) or 10% cVA (right traces) was moved, using a manual micromanipulator, within ∼0.1 mm of the antenna twice for ∼1 s, separated by a ∼4–5 s interval. The grey arrows indicate the approximate time of close approach of the cVA stimulus in wild-type and *lush* ^−/−^ sensilla. In other genotypes cVA did not evoke a response, and paraffin oil did not evoke a response in any genotype, so the precise timing of stimulation could not be determined from these traces.
(TIF)

## Abbreviations

cVA: *cis*-vaccenyl acetate
OBP: odorant binding protein
ODE: odorant degrading enzyme
OR: odorant receptor
ORCO: odorant receptor co-receptor
OSN: olfactory sensory neuron
PBP: pheromone binding protein
PDB: protein data bank
SNMP: sensory neuron membrane protein
