# Supplementary figures and images for: Ligands for Pheromone-Sensing Neurons Are Not Conformationally Activated Odorant Binding Proteins
Source: PLoS Biol. 2013 Apr 30;11(4):e1001546. doi: 10.1371/journal.pbio.1001546 (PMC3640100; doi:10.1371/journal.pbio.1001546)

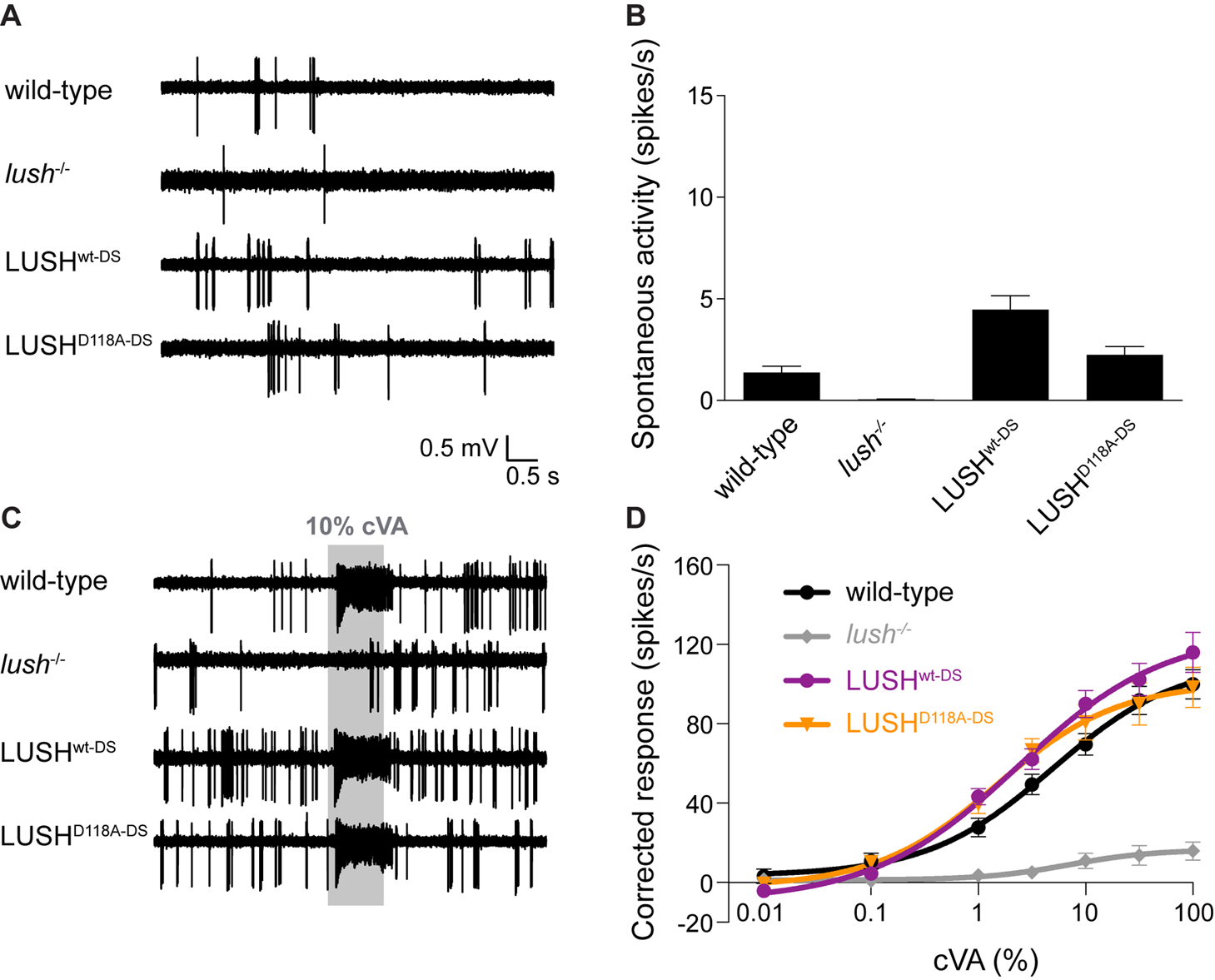

Supplement: Figure S1 — Functional analysis of an independent LUSHD118A transgene (related to Figures 4 and 5 ). (A) Representative traces of spontaneous activity in OR67d neurons in wild-type, lush −/−, LUSHwt-DS, and LUSHD118A-DS flies. (B) Quantification of mean spontaneous activity in the genotypes in (A) (±SEM; n = 19–23 sensilla; ≤3 sensilla per animal). Although we confirmed the ∼2-fold higher spontaneous activity in LUSHD118A-DS flies compared to a wild-type control, as reported [34], the firing frequency is lower than that observed in a control transgenic LUSHwt-DS strain, and falls within the range of spontaneous firing frequencies observed across our new transgenic LUSH lines (Figure 4B). (C) Representative traces of extracellular electrophysiological recordings of OR67d neurons in flies stimulated with 10% cVA in the genotypes in (A). The grey bar indicates the stimulus time (1 s). (D) Dose-response curves of OR67d neurons to cVA in the genotypes in (A). Mean responses are plotted (±SEM; n = 12–17 sensilla; ≤3 sensilla per animal). There are no statistically significant differences in cVA sensitivity due to genotype between wild-type, LUSHwt-DS, and LUSHD118A-DS animals (ANOVA, p = 0.3911). (TIF) [file pbio.1001546.s001.tif]

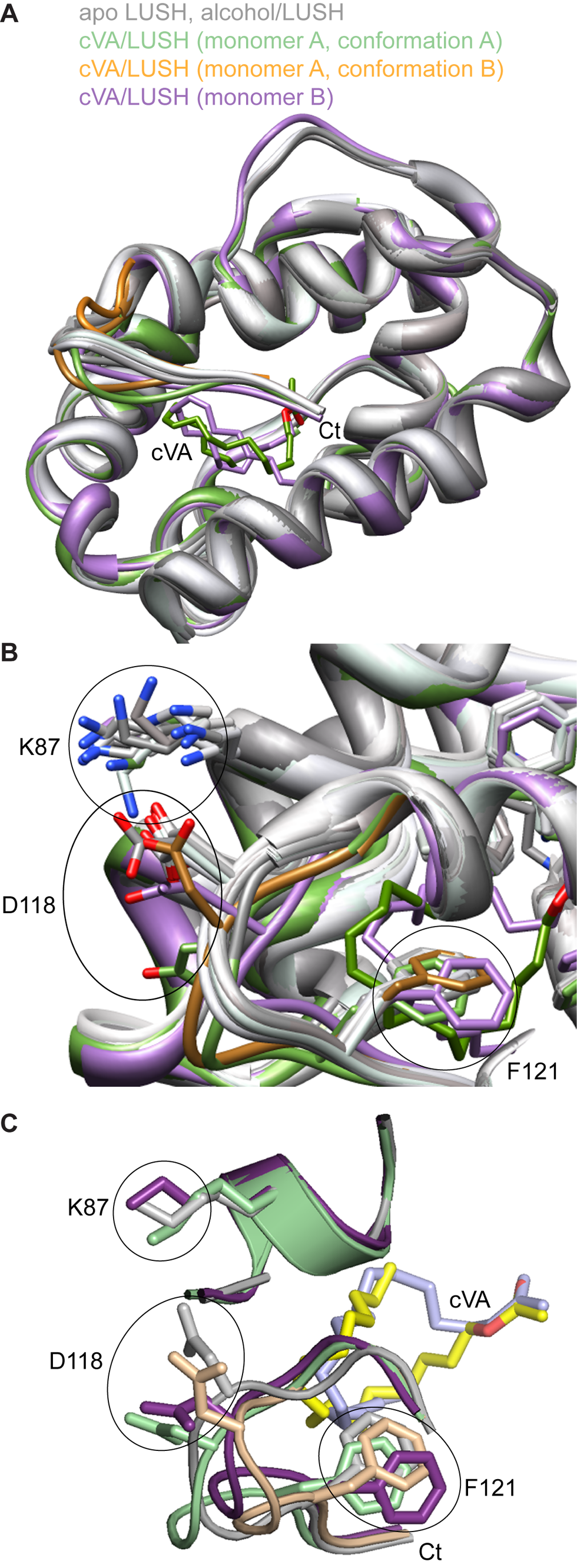

Supplement: Figure S2 — Conformational heterogeneity of LUSH crystal structures (related to Table 1 ). (A) Ribbon view of the superimposed backbones of apo LUSH, various alcohol/LUSH complexes (grey), and three conformations of the cVA/LUSH complex (green, monomer A, conformation A; beige, monomer A, conformation B; violet, monomer B). The ligand, cVA, is depicted in stick form and colored green in the monomer A structure and violet in the monomer B structure. Ct, C-terminus. (B) Close-up of the regions corresponding to residues 83–87 and 115–123 of the structures shown in (A). The side chains of K87 (nitrogen atoms colored blue), D118 (oxygen atoms colored red), and F121 are represented in stick form. Note the large diversity of side-chain conformations. (C) Close-up of residues 83–87 and 115–123 for apo LUSH (grey) and the three conformations of cVA/LUSH, colored as in (A). The side chains of K87, D118, and F121 are represented by sticks. The cVA ligand is shown in yellow (monomer A) or pale violet (monomer B). (TIF) [file pbio.1001546.s002.tif]

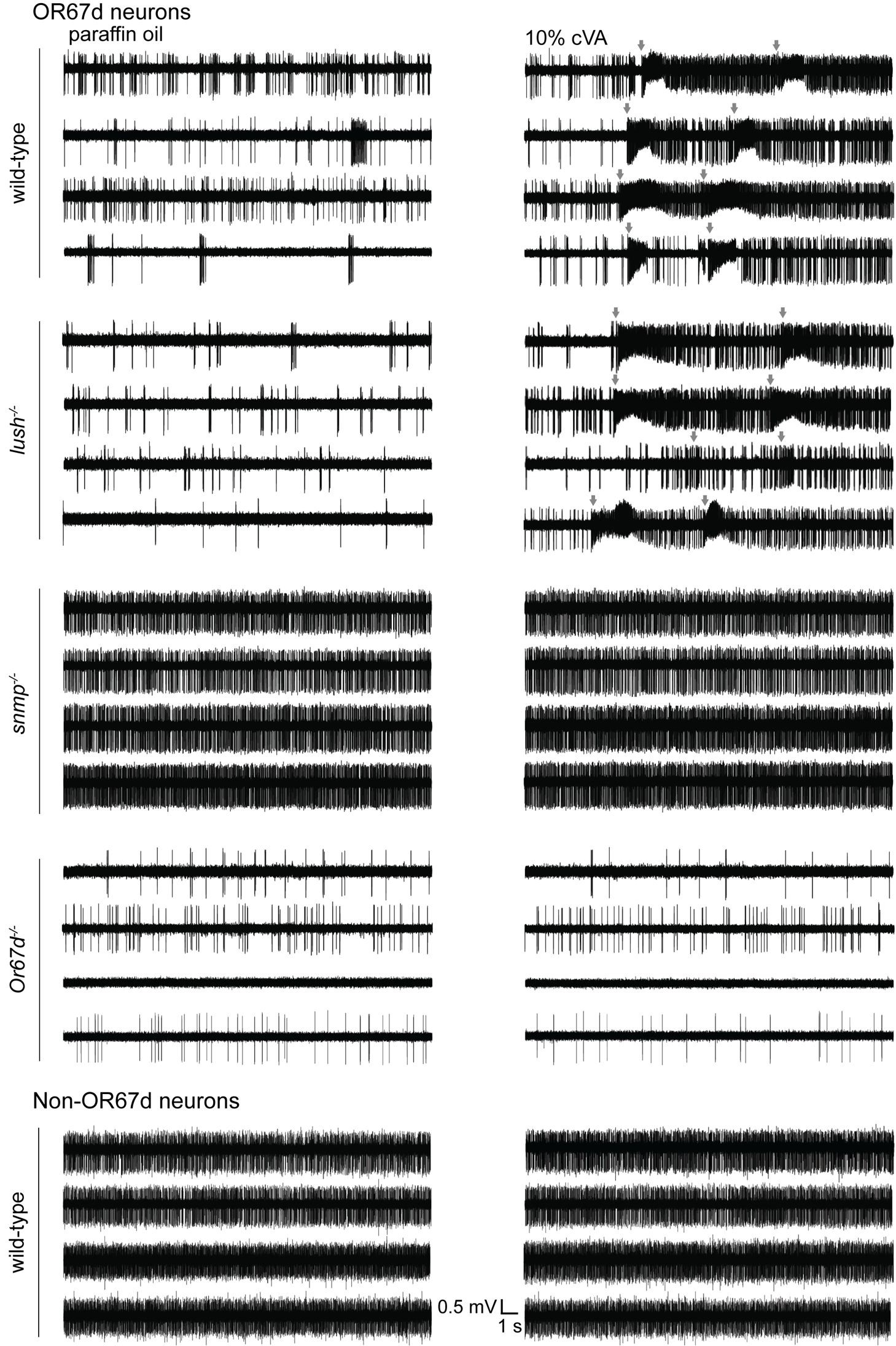

Supplement: Figure S3 — Requirement for LUSH can be bypassed by high concentrations of cVA (related to Figure 6 ). Additional traces of extracellular electrophysiological recordings of OR67d or non-OR67d neurons in the indicated genotypes stimulated in a close-range stimulation assay. During the 23 s recordings shown, a strip of filter paper spotted with paraffin oil (left traces) or 10% cVA (right traces) was moved, using a manual micromanipulator, within ∼0.1 mm of the antenna twice for ∼1 s, separated by a ∼4–5 s interval. The grey arrows indicate the approximate time of close approach of the cVA stimulus in wild-type and lush −/− sensilla. In other genotypes cVA did not evoke a response, and paraffin oil did not evoke a response in any genotype, so the precise timing of stimulation could not be determined from these traces. (TIF) [file pbio.1001546.s003.tif]
